# Retraction Note: NIR-light-mediated spatially selective triggering of anti-tumor immunity via upconversion nanoparticle-based immunodevices

**DOI:** 10.1038/s41467-026-70557-2

**Published:** 2026-03-12

**Authors:** Hongqian Chu, Jian Zhao, Yongsheng Mi, Zhenghan Di, Lele Li

**Affiliations:** 1 CAS Key Laboratory for Biomedical Effects of Nanomaterials and Nanosafety and CAS Center for Excellence in Nanoscience, National Center for Nanoscience and Technology, 100190 Beijing, China; 2 Center of Materials Science and Optoelectronics Engineering, University of Chinese Academy of Sciences, 100049 Beijing, China

Retraction to: *Nature Communications* 10.1038/s41467-019-10847-0, published online 28 June 2019

The authors have retracted this article. After publication, concerns were raised regarding a number of duplicated images in Fig. 6a, as well as Supplementary Fig. 10a and Fig. 21a. The authors checked the underlying raw data and found that these errors were introduced during the selection of representative images from the large datasets. Given the number of duplicated images, the authors have decided to retract the article.

All authors agree with this retraction.

